# Population recovery changes population composition at a major southern Caribbean juvenile developmental habitat for the green turtle, *Chelonia mydas*

**DOI:** 10.1038/s41598-019-50753-5

**Published:** 2019-10-07

**Authors:** Jurjan P. van der Zee, Marjolijn J. A. Christianen, Mabel Nava, Ximena Velez-Zuazo, Wensi Hao, Martine Bérubé, Hanneke van Lavieren, Michael Hiwat, Rachel Berzins, Johan Chevalier, Damien Chevallier, Marie-Clélia Lankester, Karen A. Bjorndal, Alan B. Bolten, Leontine E. Becking, Per J. Palsbøll

**Affiliations:** 1 Marine Evolution and Conservation, Groningen Institute for Evolutionary Life Sciences, University of Groningen, Nijenborg 7, 9747 AG Groningen, The Netherlands; 2 Wageningen Marine Research, Ankerpark 27, 1781 AG Den Helder, The Netherlands; 3 Aquatic Ecology and Water Quality Management Group, Wageningen University & Research, P.O. Box 47, 6700 AA Wageningen, The Netherlands; 4 Sea Turtle Conservation Bonaire, P.O. Box 492, Kaya Korona 53, Kralendijk, Bonaire The Netherlands; 5 Smithsonian Conservation Biology Institute, National Zoological Park, Washington, DC USA; 6 Center for Coastal Studies, 5 Holway Avenue, Provincetown, MA 02657 USA; 7 WWF Guianas, Henck Arronstraat 63, Paramaribo, Suriname; 8 ONCFS Guyane, Campus Agronomique, BP316, 97379 Kourou, French Guiana; 9 RNN Amana, Réserve Naturelle de l’Amana, Maison de la Réserve, 270 Avenue 31 Décembre, 97319 Awala-Yalimapo, French Guiana; 10 Université de Strasbourg, CNRS, IPHC, 23 Rue Becquerel, UMR, 7178 Strasbourg, France; 11 Archie Carr Center for Sea Turtle Research and Department of Biology, University of Florida, P.O. Box 118525, Gainesville, FL 32611 USA; 12 Marine Animal Ecology Group, Wageningen University & Research, P.O. Box 338, 6700 AH Wageningen, The Netherlands

**Keywords:** Conservation biology, Population dynamics, Population genetics

## Abstract

Understanding the population composition and dynamics of migratory megafauna at key developmental habitats is critical for conservation and management. The present study investigated whether differential recovery of Caribbean green turtle (*Chelonia mydas*) rookeries influenced population composition at a major juvenile feeding ground in the southern Caribbean (Lac Bay, Bonaire, Caribbean Netherlands) using genetic and demographic analyses. Genetic divergence indicated a strong temporal shift in population composition between 2006–2007 and 2015–2016 (*ϕ*_ST_ = 0.101, *P* < 0.001). Juvenile recruitment (<75.0 cm straight carapace length; SCL) from the north-western Caribbean increased from 12% to 38% while recruitment from the eastern Caribbean region decreased from 46% to 20% between 2006–2007 and 2015–2016. Furthermore, the product of the population growth rate and adult female abundance was a significant predictor for population composition in 2015–2016. Our results may reflect early warning signals of declining reproductive output at eastern Caribbean rookeries, potential displacement effects of smaller rookeries by larger rookeries, and advocate for genetic monitoring as a useful method for monitoring trends in juvenile megafauna. Furthermore, these findings underline the need for adequate conservation of juvenile developmental habitats and a deeper understanding of the interactions between megafaunal population dynamics in different habitats.

## Introduction

Different populations of the same species of migratory megafauna may depend on the same key habitats during parts of their life cycle. Developmental habitat shifts between successive life stages result in the mixing of offspring from different breeding populations in juvenile feeding grounds in many marine taxa including fishes^1^ and sea turtles^2^. Mixed aggregations in key developmental habitats present unique challenges to conservation and management because anthropogenic impacts potentially affect the future generations of multiple populations^2^. The conservation risk of population overlap is exacerbated for vulnerable populations, such as small populations of threatened species. Understanding the dynamics and composition at key developmental habitats, such as juvenile feeding grounds, is thus of paramount concern to conservation and management^2^.

However, little is known about the temporal dynamics at key developmental habitats even though recruitment is unlikely to remain constant^3–5^. Dispersal of marine species is governed by different processes at different life stages. Ocean currents influence dispersal of early life stages and temporal fluctuations in ocean currents can produce changes in the population composition at juvenile feeding grounds^3,4^. In addition, juvenile recruitment to feeding grounds can fluctuate over time due to environmental stochasticity affecting the production and survival of offspring^4^ as well as long-term declines in reproductive success^5^. Human activities too can cause negative and positive changes, both through destructive activities as well as conservation measures. In the extreme case that human activities result in the local extinction of breeding populations, these extirpated populations will no longer produce recruits. By contrast, increases in reproductive success, for example due to conservation measures, can enhance recruitment^6^ and possibly change the dynamics at juvenile feeding grounds.

A prime example of where the dynamics and composition at juvenile feeding grounds could be altered by conservation measures are green turtles (*Chelonia mydas*). Past human exploitation and habitat degradation has decimated sea turtle populations globally^7–9^. In the Caribbean, the present abundance of green turtles is at less than one percent of pre-exploitation levels based upon historical data^9^. Recent studies have highlighted that some rookeries are showing signs of population recovery following past sea turtle conservation measures^10–14^. Protection of nesting females has led to substantial increases in nesting trends, i.e. the number of nests produced, in several rookeries^10,12,13^. Increasing nesting trends as high as 14% per year have been reported in rookeries in Florida^10,14^. Rookeries in the eastern Caribbean seemed to have recovered at a slower pace compared to the north-western Caribbean^13,14^. Adult female abundance at the Aves Island, Venezuela, rookery has been increasing by approximately 5% per year during recent decades^13^. Little is known, however, what effects this population recovery may have on the dynamics and composition at juvenile feeding grounds.

In sea turtles, hatchlings disperse from natal rookeries to oceanic developmental habitats through a combination of active swimming and passive drifting in ocean currents^15,16^. Juvenile sea turtles later recruit to coastal feeding grounds^17^ shared by multiple rookeries. Juvenile dispersal is influenced by factors, such as; ocean currents^18^, distance among rookeries and feeding grounds^18^, natal homing^19^ and adult female abundance at rookeries^20^. Given the influence of abundance^20^ and reproductive output at rookeries^4,5^ upon sea turtle dispersal, it is possible that the population composition at juvenile feeding grounds changes as a result of differences in rookery recovery rates.

The hypothesis that the population composition at juvenile feeding grounds changes due to differential recovery rates can be tested by studying temporal genetic heterogeneity at feeding grounds using mitochondrial DNA (mtDNA). There is strong population structure in mtDNA diversity among sea turtle rookeries as a result of natal homing in adult females^21^. MtDNA markers can therefore be used as genetic tags to estimate juvenile recruitment into a feeding ground by assessing the relative proportion different rookeries contribute to a juvenile feeding ground (e.g.^18,20^). If recovery rates differ among genetically diverged rookeries, temporal genetic heterogeneity is expected to increase with time, i.e. reflecting a directional change in mtDNA diversity. However, investigating changes in recruitment over longer timeframes is warranted because juveniles can spend up to 15 years at feeding grounds before moving to deeper feeding grounds (e.g.^22,23^), which could lead to autocorrelation in mtDNA diversity between successive years. Autocorrelation in mtDNA diversity can be further diminished by investigating changes in recruitment using only recent recruits, i.e. small juveniles that presumably arrived recently at a feeding ground.

The present study assessed the relationship between differential recovery rates and changes in the population composition at a major juvenile green turtle feeding ground^24^ located in Lac Bay (Bonaire, Caribbean Netherlands) in the southern Caribbean. Decadal changes in population composition were investigated by estimating temporal genetic heterogeneity and assessing changes in juvenile recruitment between 2006–2007 and 2015–2016. Decadal changes in juvenile recruitment were correlated with rookery recovery rates that were estimated as the product of population growth rates and adult female abundance to account for variation in population size among rookeries. The genetic and demographic analyses were conducted at both the level of individual rookeries and rookeries grouped into three regions reflecting mtDNA stocks recognized in sea turtle conservation and management^25^.

## Results

### Genetic diversity and temporal genetic structure

**Figure 1:**
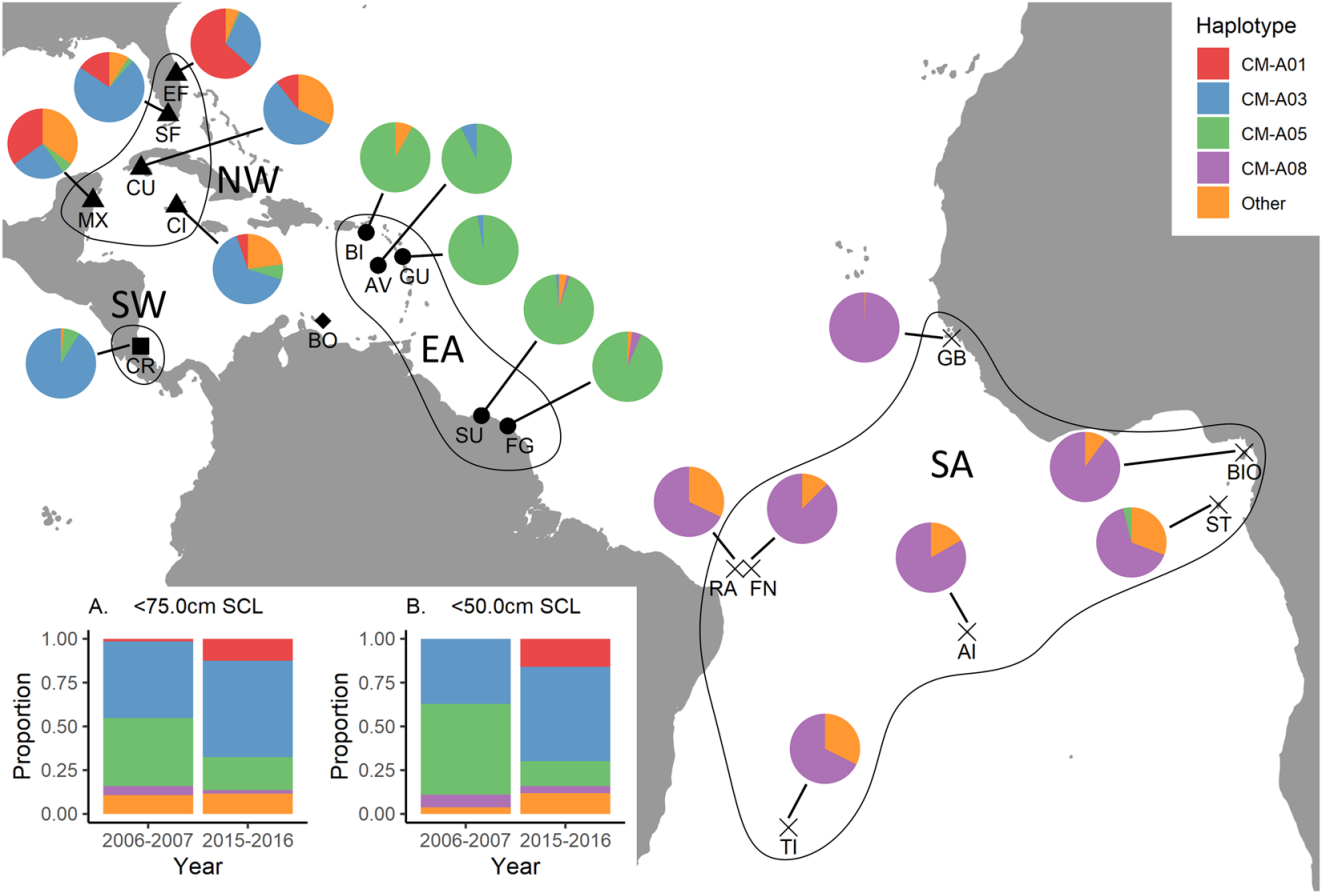
Common Atlantic mtDNA haplotype proportions (CM-A01, CM-A03, CM-A05 and CM-A08; remaining haplotypes pooled under ‘Other’) in Lac Bay, Bonaire (BO; diamond) in 2006–2007 and 2015–2016 for <75.0 cm SCL juveniles (bottom-left insert (**A**)) and <50.0 cm SCL juveniles (bottom-left insert (**B**)) and in north-western (NW; triangles), south-western (SW; square), eastern (EA; circles) Caribbean and southern Atlantic (SA; crosses) green turtle rookeries: Aves Island, Venezuela (AV)^47,70^; Buck Island, St. Croix, US Virgin Islands (BI)^47^; Cayman Islands (CI)^74^; Cuba (CU)^71^; Tortuguero, Costa Rica (CR)^40,70^; Central Eastern Florida, USA (EF)^72^; French Guiana (FG)^73^; Guadeloupe (GU)^73^, Quintana Roo, Mexico (MX)^70^; South Florida, USA (SF)^72^; Suriname (SU)^47,70,73^; Rocas Atoll, Brazil (RA)^70,76^; Fernando de Noronha, Brazil (FN)^76^; Trindade Island, Brazil (TI)^76^; Ascension Island, UK (AI)^70,75,79^; Poilão, Guinea Bissau (GB)^70,75,77^; São Tomé and Príncipe (ST)^75^; Bioko Island, Equatorial Guinea (BIO)^75^.

The sequence of 474 base pairs of the mtDNA control region was determined in 332 juvenile green turtles (30.0–75.0 cm maximum straight carapace length (SCL); mean ± SD = 50.66 ± 9.71 cm) sampled in Lac Bay between 2006 and 2016 (Tables S1–S2). Re-sequencing revealed one discrepant mtDNA sequence, corresponding to a sequencing consistency rate at >99%. Nineteen mtDNA haplotypes were detected among the 332 mtDNA sequences (Tables S1–S2), including a previously unreported mtDNA haplotype (GenBank accession number: MN481527; Fig. S1) designated ‘CM-A76’ in accordance with the commonly employed Atlantic green turtle mtDNA haplotype nomenclature, e.g.^26^. Adding 41 recaptured individuals increased the final sample size to 373 mtDNA control region sequences. The most common mtDNA haplotypes were CM-A03 (52%) and CM-A05 (27%) followed by CM-A01 (9%) and CM-A08 (3%; Table S1). Haplotypes CM-A01 and CM-A03 increased in frequency during the study period while the frequency of CM-A05 decreased (Fig. 1 insert). The number of haplotypes and haplotype diversity varied among years without any apparent temporal trend (Table S2). Nucleotide diversity was lower during recent years (e.g. *π* = 0.011 in 2006–2007 and *π* = 0.008 in 2015–2016; Table S2). A significant degree of genetic divergence was detected between 2006–2007 and 2015–2016 (*ϕ*_ST_ = 0.101, *P* < 0.001). A significant and positive correlation was detected between pairwise estimates of genetic divergence among years (Fig. S2; Table S4) and time (Mantel test, *P* = 0.038, *r*^2^ = 0.12). Including only <50.0 cm SCL juveniles in the analysis increased the genetic divergence between 2006–2007 and 2015–2016 (*ϕ*_ST_ = 0.271, *P* < 0.01) and resulted in a stronger correlation between genetic divergence (Fig. S3; Table S5) and time (*P* = 0.012, *r*^2^ = 0.21). However, the elevated genetic divergence could be due to sampling variance and smaller sample sizes (Table S3).

### Temporal changes in juvenile recruitment

**Figure 2:**
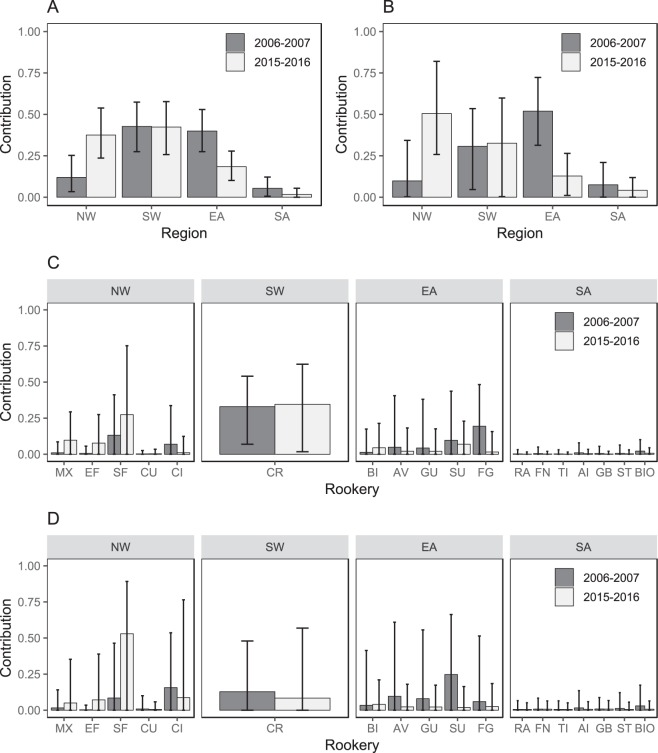
Mean and 95% CI of the estimated contribution to the Lac Bay juvenile green sea turtle feeding ground in 2006–2007 and 2015–2016 per region for (**A**) <75.0 cm SCL and (**B**) <50.0 cm SCL juveniles, and per rookery for (**C**) <75.0 cm SCL and (**D**) <50.0 cm SCL juveniles. Rookeries are grouped by region. Abbreviations are described in Fig. 1.

Temporal changes in juvenile recruitment were observed for the eastern (Δ*C* = −0.22) and north-western Caribbean (Δ*C* = 0.26; Fig. 2A; Table 1). The contribution from the north-western Caribbean region to the Lac Bay juvenile feeding ground increased from 12% in 2006–2007 to 38% in 2015–2016. By contrast, the contribution from the eastern Caribbean decreased from 40% (2006–2007) to 18% (2015–2016). Juvenile recruitment from the south-western Caribbean region appeared constant throughout the study period (Δ*C* = 0.02; Fig. 2A; Table 1). Overall recruitment from the southern Atlantic was low (2006–2007: 5%; 2015–2016: 2%; Fig. 2A) and showed a slightly decreasing trend (Δ*C* = −0.04). Estimates of Δ*C* were generally close to zero for individual rookeries, with the exception of French Guiana (Δ*C* = −0.18) and southern Florida (Δ*C* = 0.44). Rookery contributions were estimated with wide uncertainties for individual rookeries (Fig. 2C; Table 1). The mean contribution of the Cayman Islands was higher than expected (2006–2007: 7%; 2015–2016: 1%) given the low abundance of adult females at this rookery^27^, but this is partially explained by the unweighted prior used in the mixed stock analysis.

**Table 1:** Weighted mean annual population growth rate (*r*), adult female abundance (*N*), minimum geographic distance to Lac Bay, Bonaire (*D*), expected reproductive output (*Nr*) and temporal changes in recruitment for <75.0 cm SCL and <50.0 cm SCL juveniles (Δ*C*) per region and rookery. Region is shown for each rookery.

Name	Region	*N*	*D*	*r*	*Nr*	∆*C*_<75.0cm_	∆*C*_<50.0cm_
North-western Caribbean (NW)	—	29003	1629	0.136	3944	0.26	0.41
South-western Caribbean (SW)	—	131751	1696	0.017	2240	0.00	0.02
Eastern Caribbean (EA)	—	22013	1009	0.066	1448	−0.22	−0.39
Southern Atlantic (SA)	—	46020	5985	0.035	1610	−0.04	−0.03
Mexico (MX)	NW	18257	2142	0.139	2538	0.09	0.03
Central Eastern Florida (EF)	NW	4990	1434	0.183	913	0.07	0.07
Southern Florida (SF)	NW	3314	1483	0.100	333	0.14	0.44
Cuba (CU)	NW	2226	1707	0.122	270	0.00	0.00
Cayman Islands (CI)	NW	72	1377	0.056	4	−0.06	−0.07
Costa Rica (CR)	SW	131751	1696	0.017	2240	0.02	−0.04
Buck Island (BI)	EA	63	475	0.056	4	−0.01	−0.02
Aves Island (AV)	EA	2833	538	0.045	127	−0.03	−0.07
Guadeloupe (GU)	EA	50	767	0.056	3	−0.02	−0.06
Suriname (SU)	EA	13067	1508	0.082	1065	−0.03	−0.23
French Guiana (FG)	EA	6000	1756	0.041	248	−0.18	−0.03
Rocas Atoll (RA)	SA	275	3965	0.024	7	0.00	0.00
Fernando de Noronha (FN)	SA	70	4115	0.035	2	0.00	0.00
Trindade Island (TI)	SA	2016	4825	0.035	71	0.00	0.00
Ascension Island (AI)	SA	13417	6161	0.043	577	−0.01	−0.01
Guinea Bissau (GB)	SA	29016	5849	0.035	1015	0.00	0.00
São Tomé and Príncipe (ST)	SA	376	8382	0.035	13	0.00	−0.01
Bioko Island (BIO)	SA	850	8596	−0.088	−75	0.03	0.01

Including only <50.0 cm SCL juveniles in the mixed stock analysis resulted in stronger increased recruitment from the north-western Caribbean (10% to 50%; Δ*C* = 0.41) and decreased recruitment from the eastern Caribbean (52% to 13%; Δ*C* = −0.39) between 2006–2007 and 2015–2016 (Fig. 2B; Table 1). These trends were also reflected in contributions estimated at the level of rookeries (Fig. 2D). Overall recruitment from the south-western Caribbean was stable but lower for <50.0 cm juveniles (Δ*C* = 0.02; 31% in 2006–2007 and 33% in 2015–2016; Fig. 2D). However, there was also greater uncertainty in regional- and rookery contribution estimates.

### Correlation with rookery recovery trends

The highest population growth rate (*r* = 0.136) and reproductive output (*Nr* = 3,944) was estimated for the north-western Caribbean region (Table 1). Lower population growth rates were estimated for the eastern Caribbean (*r* = 0.066), south-western Caribbean (*r* = 0.017) and southern Atlantic (*r* = 0.035) regions. Nesting trends in Suriname were not linear but seemed to increase mainly during the last decade (Table S6). The expected reproductive output of the south-western Caribbean (*Nr* = 2,240) was higher than that estimated for the eastern Caribbean (*Nr* = 1,448) due to the high adult female abundance at the Tortuguero rookery in Costa Rica (Table 1). The scarcity of rookery-specific data necessitated assuming regional population growth rate estimates for the Buck Island, Guadeloupe, Fernando de Noronha, Trindade Island, Bioko Island and São Tomé and Príncipe rookeries.

Spearman’s correlation coefficient *ρ* was estimated at 1.00 (*P* = 0.083) at the regional level for both <75.0 cm SCL and <50.0 cm SCL juveniles. The monotonic association between Δ*C* and *Nr*, i.e. the ranking of Δ*C* and *Nr* matched perfectly at the regional level but was nearly significant, which was probably the consequence of only four data points that resulted from grouping rookeries into regions. At the level of rookeries, the monotonic association between Δ*C* and *Nr* was non-significant for both <75.0 cm juveniles (*ρ* = 0.46, *P* = 0.16) and <50.0 cm juveniles (*ρ* = 0.13, *P* = 0.71). However, logistic regression suggested *Nr* was a nearly significant predictor for whether recruitment increased or decreased for <75.0 cm SCL juveniles (*P* = 0.09), but not for <50.0 cm SCL juveniles (*P* = 0.25).

A linear model incorporating the natural logarithm of adult female abundance *N* (*P* = 0.047) and geographical distance *D* (*P* = 0.094) was found to best describe mean rookery contribution estimates in 2006–2007 for <75.0 cm SCL juveniles (Table S7). By contrast, a linear model that only incorporated *Nr* (*P* = 0.012) was best-supported in 2015–2016 (<75.0 cm). However, a model incorporating only *D* (*P* = 0.066) represented the best fit in 2006–2007, while the null model (i.e. no predictors) was best-supported in 2015–2016. Though *D* was non-significant at an *α* of 0.05 in the best-supported models in 2006–2007 for both size classes, this was likely an effect of the relative small number of data points (i.e. 18 rookeries) in the multiple linear regression. In addition, excluding *D* resulted in poorer model performance (Table S7).

## Discussion

The present study assessed the changes in population composition at a major juvenile green turtle feeding ground located in Lac Bay, Bonaire, during the last decade. Genetic and demographic analyses suggested an increase in the proportion of juvenile green turtles in Lac Bay from rapidly recovering rookeries in the north-western Caribbean during the study period. In the north-western Caribbean, recovery of rookeries has previously been associated with increases in juvenile abundance at local, proximate feeding grounds^6^. However, juvenile green turtle abundance did not increase significantly in Lac Bay during the last decade^28^. If abundance were stable, the observed temporal changes in juvenile recruitment to the Lac Bay feeding ground would have reflected changes in the abundance of juveniles contributed by different Caribbean rookeries. If this presumption is correct, past sea turtle conservation measures in the north-western Caribbean could have resulted in increased juvenile abundance even at distantly located feeding grounds. By contrast, fewer juveniles originated from the eastern Caribbean and southern Atlantic. The decreased juvenile recruitment from the eastern Caribbean could be an early warning signal that reproductive output is declining in that region^29^. In the northern Great Barrier Reef, increased tidal inundation and rainwater flooding have been associated with reduced hatching success at the Raine Island green turtle rookery and decreased juvenile recruitment to local feeding grounds^5^. Rookeries in the southern Great Barrier Reef have been recovering during the last few decades^10^ also suggested a potential effect of population recovery on juvenile recruitment^5^, though a lack of nesting trend data from rookeries outside the Great Barrier Reef precluded investigating this hypothesis^5^.

However, differences in nesting trends at rookeries and recruitment to juvenile feeding grounds are difficult to interpret^29^. Juvenile sea turtles form mixed aggregations at feeding grounds comprised of individuals from many different rookeries^18,20^ that can differ in nesting trends^10,13,14^. Furthermore, adult females within a rookery will often utilize different geographically disparate feeding grounds^30^. Differential feeding habitat use can contribute to skewed contributions to reproductive success^31^. Hatchling mortality and nest production vary considerably between successive years and can lead to interannual fluctuations in juvenile recruitment to feeding grounds^4,32^. Recruitment to juvenile feeding grounds may also depend on population densities relative to carrying capacities^29^. Dynamics at juvenile feeding grounds are also expected to lag behind changes in nesting trends due to the time between hatching and recruiting to coastal feeding grounds^29^. However, more long-term studies will be required to further understand the time lag between hatching and recruitment, and its effect on metapopulation dynamics.

The decreased recruitment from the eastern Caribbean can possibly be explained by a difference in the timing of nesting trends. Nesting trends were stable in Suriname, the largest green turtle rookery in the eastern Caribbean, between the 1970’s and the 2000’s and started to increase during the last decade, while nesting trends in the Archie Carr National Wildlife Refuge green turtle rookery in Florida increased since the 1990’s^10^. Green turtles spend approximately 3 to 5 years in oceanic habitats^17^ before recruiting to coastal feeding grounds at a SCL of 25 to 35 cm^23^. Juveniles arrive in Lac Bay at 35 to 40 cm (SCL) at an age of 6 to 10 years depending on their rate of growth^6,33^. Changes in juvenile recruitment to Lac Bay are therefore expected to lag up to a decade behind changes in nesting trends. Given that nesting trends in Suriname have started to increase during the last decade, it is possible that juvenile recruitment from the eastern Caribbean will increase in the near-future. This does not explain why recruitment from the eastern Caribbean decreased despite long-term stability in nesting trends. However, nesting trends may be a poor proxy for reproductive output. For example, hatching success may be very sensitive to environmental fluctuations, such as changes in sand temperature at nesting beaches^34^. Nesting trends may therefore appear stable, but nonetheless result in few recruits due to low hatching success.

Sea turtles may shift to other feeding grounds between different size classes as a result of juvenile natal homing^19,35,36^. This was recently demonstrated in a study of juvenile green turtles in Japanese feeding grounds, where the contribution of local rookeries was higher for larger (i.e. 50–70 cm SCL) juveniles^35^. In the present study, recruitment differed between the two size partitions (i.e. <50.0 cm and <75.0 cm) within 2006–2007 and 2015–2016. Recruitment from the south-western Caribbean was higher overall when all juveniles were analysed, while recruitment from the north-western and eastern Caribbean was higher for small juveniles. These differences are potentially due to juvenile natal homing, reflecting the emigration of larger juveniles of north-western or eastern Caribbean origins from Lac. In addition, this implies that studying the recruitment dynamics of small juveniles is warranted to understand the link between population dynamics at feeding grounds and rookeries if larger juveniles shift between feeding grounds.

The stable abundance of green turtles^28^ raises the possibility that the Lac Bay feeding ground is at or near carrying capacity. Species can respond to increased densities by dispersing to other feeding grounds^37^ and it has been argued that dispersal in green turtles may in part be density-dependent^33^. Increased abundances at feeding grounds may lead to an increased propensity to disperse as local abundance nears carrying capacity and competition for resources increases^6,33^. For example, the exceeding of local carrying capacity in a juvenile feeding ground in the Bahamas resulted in net emigration of individuals over subsequent years until abundance stabilized^33^. If a feeding ground is at carrying capacity and dispersal propensity is equal for all individuals within a feeding ground, the equilibrium population composition is expected to be determined by the relative levels of recruitment from various source populations. Population composition may therefore change as a result of recruitment from one rookery outweighing recruitment from another rookery. In other words, rookeries contributing a larger number of offspring to a feeding ground may increasingly dominate shared feeding grounds over time, akin to a ‘displacement effect’. This displacement effect could in part explain decreased recruitment despite stable nesting trends in the eastern Caribbean. Further monitoring of juvenile recruitment and abundance at feeding grounds in relation to population dynamics and reproductive output at rookeries is warranted to investigate whether such a displacement effect occurs.

Short-term fluctuations in mtDNA diversity between successive years in sea turtles can be a result of stochasticity in reproductive output^4^. By contrast, if long-term effects such as declines^5^ or increases in hatching success, lead to differential juvenile recruitment to feeding grounds over time, genetic signals of ‘directional change’ in mtDNA diversity over multiple years, e.g. a decade, are expected. Short-term and long-term effects can therefore possibly be disentangled by assessing the direction and nature of changes in genetic heterogeneity during longer time intervals, as was done in the present study. The degree of temporal genetic heterogeneity observed during this study correlated strongly with time and suggested a directional change in mtDNA diversity. The temporal genetic heterogeneity we observed in Lac Bay was similar to levels recorded among different green turtle feeding grounds (e.g. *ϕ*_ST_ = 0.168 between Barbados and Almofala in northern Brazil^38^). These findings suggest the observed temporal genetic heterogeneity is in part explained by long-term changes in juvenile recruitment though the presence of short-term effects could not be rejected. However, short-term and long-term effects are not necessarily mutually exclusive^4^.

Genetic changes within rookeries, e.g. via genetic drift^39^, during the study period could, in theory, lead to a false-positive signal of juvenile recruitment. However, this is unlikely to explain the findings in the present study since no temporal heterogeneity in mtDNA has been observed at any rookeries so far^40–42^, although these studies only lasted two to three years. Temporal heterogeneity in mtDNA diversity has been reported at a loggerhead sea turtle rookery in Florida but could simply be due to sampling variance^43^. Second, possible changes in mtDNA diversity at the regional level during the study would require gene flow among distant rookeries which is unlikely given the high degree of natal homing to rookeries observed in nesting female sea turtles^21^.

Genetic assignment methods such as mixed stock analysis have reduced statistical power to determine the origin of individuals when putative sources are genetically similar^44^. Green turtle rookeries in the Caribbean differ substantially in mtDNA haplotype composition at the regional level but less so within regions. Accordingly, the estimated contribution from individual Caribbean rookeries was subject to a high degree of uncertainty in this study and complicated making inferences at the level of individual rookeries. Sequencing longer fragments of the mtDNA control region^45^, mitochondrial short tandem repeats^46^ or mitochondrial genomes^47^ could possibly reduce uncertainties in mixed stock analysis. However, this would require generating novel genetic data for all major Caribbean green turtle rookeries, which was beyond the scope of this study.

Effective conservation measures are key in an era characterized by an accelerated loss of biodiversity driven by anthropogenic activities. Sea turtle conservation and management has typically focused upon protecting nesting beaches^48^, even though nesting beaches constitute only a small, albeit vital, part of the life history of sea turtles. The increasing numbers of juveniles originating from rookeries that are showing signs of recovery observed in the present study are encouraging and highlights the success of current attention to sea turtle nesting beaches. In addition, the present study demonstrates genetic monitoring may represent a useful method for monitoring trends in juveniles, which may provide an early warning signal for declining reproductive success^29^ and improve our understanding of sea turtle metapopulation dynamics^48^. However, sufficient feeding habitat quality and quantity is required for juveniles to mature and contribute to future generations^48^, and fortunately feeding habitats are increasingly receiving attention^49^. Climate change, invasive species and habitat degradation continue to threaten developmental habitats of sea turtles^50–52^. In Lac Bay, the invasive seagrass *Halophila stipulacea* has expanded rapidly during recent years^53^ and a recent study demonstrated green turtles facilitate the expansion of *H*. *stipulacea* through selectively grazing on the native seagrass *Thalassia testudinum*^28^. If developmental habitat quality and quantity are not maintained, increased abundances of juveniles may elevate intra-specific competition for resources and increase risks of overconsumption, e.g. overgrazing of seagrass meadows^54^, and habitat collapse^55^. An understanding of the interactions between nesting trends at rookeries, recruitment and dynamics at feeding grounds as well as an understanding of the ecological interactions within feeding grounds is required to ensure adequate protection of both adult breeding and juvenile developmental habitats in endangered marine megafauna.

## Methods

All fieldwork was conducted under ‘Openbaar Lichaam Bonaire’ permit nr. 558/2015-2015007762 granted by the ‘Executive Council of the Public Entity of Bonaire’ to Sea Turtle Conservation Bonaire (STCB) in accordance with the required animal care protocols. As a member of the Wider Caribbean Sea Turtle Network (WIDECAST), STCB uses best-practice standardized protocols for sampling and handling sea turtles. Tissue samples originated from juvenile green turtles (maximum straight carapace length (SCL) below 75.0 centimetres (cm)). Green turtles were captured by hand or nets in Lac Bay in Bonaire between 2009 and 2016. Tissue samples were collected from the dorsal neck epidermal area using a sterilized scalpel blade or a 6 mm biopsy punch (Integra™ Miltex®), preserved in 6 M sodium chloride with 25% dimethyl sulfoxide^56^ or in 70% ethanol, stored locally at −4 degrees Celsius (°C) and archived at −20 °C upon arrival at the University of Groningen, the Netherlands. Total-cell DNA was extracted using the Gentra Puragene® Tissue Kit (QIAGEN Inc.) following the manufacturer’s instructions.

A 474 base pair fragment of the mitochondrial DNA control region was amplified by nested PCR (polymerase chain reaction) amplifications^57,58^. An initial PCR amplification was conducted using the primers CM15412F (forward; 5′-AAAGCATTGGTCTTGTAAACC-3′) and CM16333R (reverse; 5′-TATGTCAGTTTGGTCAGTCTC-3′) followed by a PCR amplification using the primers CM15791F (forward; 5′-CAACCATGAATATTGTCACAGT-3′) and CM15984R (reverse; 5′-CATTCAACCAAAGGCCTTTTA-3′). PCR amplifications were conducted in a 10 μL reaction volume containing 1 μM of each primer, 1X standard *Taq* reaction buffer (New England Biolabs® Inc.), 0.2 μM of each dNTP, 0.4 units of *Taq* DNA polymerase (New England Biolabs® Inc.), autoclaved Milli-Q® H_2_O and between 5 to 15 ng extracted DNA. The PCR conditions consisted of an initial cycle of 2 minutes at 94 °C followed by 32 cycles each of 30 seconds at 94 °C, 45 seconds at 55 °C and 30 seconds at 72 °C, and a single final step of 5 minutes at 72 °C. Excess primers and nucleotides were removed from the amplifications prior to cycle-sequencing by addition of one unit of shrimp alkaline phosphatase (FastAP™, Thermo Fisher Scientific Inc.) and five units of exonuclease I (New England Biolabs® Inc.) as described by Werle *et al*.^59^. Cycle-sequencing was performed with primers CM15412F (for the first fragment) and CM15791F (for the second fragment) using an ABI BigDye® Terminator v3.1 Cycle Sequencing Kit (Applied Biosystems Inc.) following the manufacturer’s instructions but using 1/16 of the BigDye® Ready Reaction Mix. The PCR conditions consisted of 25 cycles each with 10 seconds at 96 °C, 5 seconds at 50 °C and 4 minutes at 72 °C. Excess nucleotides and primers were removed by ethanol/EDTA precipitation. The cycle-sequencing products were re-suspended overnight in deionized formamide and the order of cycle-sequencing products was resolved by capillary electrophoresis on an Applied Biosystems 3730xl DNA Analyzer™ (Life Technologies Inc.) at the University of Groningen.

MtDNA control region consensus sequences were assembled from one or more DNA sequences per DNA extraction using a custom software pipeline (Palsbøll, *unpublished*). Individual mtDNA sequences from each sample were assembled, the consensus sequence estimated from the Phred quality scores and subsequently aligned to a mtDNA control region green turtle reference sequence (GenBank accession number: JN632497.1) and truncated to 474 bp using MIRA ver. 4.9.5.2^60^. Assembled sequences were aligned with MUSCLE ver. 3.8.31^61^ in SEQOTRON ver. 1.0.1^62^ using the default parameter settings. Aligned sequences were visually inspected and manually corrected. A total of 96 randomly chosen DNA extractions were re-sequenced to assess sequencing consistency rates.

The data generated in the present study were combined with mtDNA sequence haplotype frequency data from juvenile green turtles (SCL < 75.0 cm) sampled in Lac Bay during 2006 and 2007^63^. Two temporal sample partitions denoted ‘2006–2007’ and ‘2015–2016’ comprised individuals sampled in 2006 and 2007, and in 2015 and 2016. An additional sample partition comprised all data partitioned according to sampling years where recaptured individuals were accounted for by treating them as additional observations, i.e. an individual first captured in 2007, recaptured (and sampled) in 2010 and recaptured again in 2015 was represented as three observations of the same individual, one in each observed year.

MtDNA haplotype diversity^64^ and nucleotide diversity^64^ and genetic divergence (*ϕ*_ST_^65^) between 2006–2007 and 2015–2016 and among years was estimated using ARLEQUIN ver. 3.5.2.2^66^. The statistical significance of *ϕ*_*ST*_ was estimated from 10,000 random permutations of the data. The correlation between genetic divergence and temporal distance, measured in years, was estimated using a Mantel test^67^ as implemented in ARLEQUIN^68^. Population genetic analyses were repeated using only recent recruits (i.e. small juveniles with SCL-max <50.0 cm).

Temporal changes in juvenile recruitment were inferred estimating the contribution of Caribbean rookeries (Fig. 1) to the Lac Bay turtle feeding ground in 2006–2007 and 2015–2016 using the Bayesian mixed stock analysis approach implemented in BAYES ver. 11/23/11^69^. Published mtDNA control region haplotype (also 474 bp) data from Atlantic green turtle rookeries^40,47,70–77^ were used as source populations (Appendix 1). Two mixed stock assessments were conducted: (1) rookeries were grouped into regions (Fig. 1); ‘north-western Caribbean’, ‘south-western Caribbean’, ‘eastern Caribbean’^20^ and ‘southern Atlantic’ and (2) individual rookeries representing source populations. Additional mixed stock assessments were conducted using only small juveniles (<50.0 cm SCL) that presumably represent recent recruits and are more informative of recent changes in recruitment dynamics. A uniform prior was used where the prior contribution was equal among each source, i.e. prior contribution was set to 1/*k* where *k* was the number of putative sources, to avoid bias in subsequent demographic analyses. The employed MCMC settings are listed in Table S8. A Gelman-Rubin shrink factor below 1.2 was inferred as MCMC chain convergence^69^. Temporal changes in juvenile recruitment were estimated as:

1 $$\Delta C={C}_{j}-{C}_{i}$$

where *C*_*i*_ was the mean contribution estimated for 2006–2007 and *C*_*j*_ was the mean contribution estimated for 2015–2016.

Annual population growth rates at north-western, south-western Caribbean rookeries were estimated by calculating the weighted mean of annual population growth rates at nesting sites (data from Mazaris *et al*.^14^) within rookeries (Tables S9-S10). Nest site-specific annual population growth rates were weighted using estimates of adult female abundance at nesting sites^27,73^. For the eastern Caribbean, annual population growth rates at the French Guiana and Suriname rookeries were estimated using temporal trends in the number of recorded nests (Table S6) using the approach adopted from Mazaris *et al*.^14^:

2 $$r={(\frac{{N}_{L}}{{N}_{F}})}^{(\frac{1}{n-3})}-1$$

where *r* is the annual population growth rate, *N*_*L*_is the mean number of recorded nests in the last three years of the time series, *N*_*F*_ the mean number of recorded nests in the first three years of the time series and *n* the length of the time series. The estimate of the annual population growth rate at the Aves Island rookery was obtained from García-Cruz *et al*.^13^. Southern Atlantic rookery annual population growth rates were obtained from Mazaris *et al*.^14^ (Bioko Island and Rocas Atoll) and Weber *et al*.^11^ (Ascension Island). Regional annual population growth rates were estimated as weighted mean annual population growth rates for rookeries within regions.

The product of the annual population growth rate *r*and the adult female abundance *N* (*Nr*) was used as a proxy for reproductive output in order to account for variation in adult female abundance among rookeries. The association between Δ*C* and *Nr*, was tested using nonparametric Spearman rank-order correlation in R ver. 3.5.3^78^. In addition, we tested how well *Nr* predicted whether recruitment increased or decreased over time at the level of rookeries (i.e. as a binary response variable) using logistic regression in R ver. 3.5.3. Multiple linear regression was used to test the effect of *N*, geographic distance (*D*; estimated as the shortest distance between source populations and the study site using the *geosphere* R package ver. 1.5–10) and *Nr* on mean rookery contribution estimates during 2006–2007 and 2015–2016 for both size classes. Model selection was performed via a stepwise algorithm using the Akaike Information Criterion (AIC) in R ver. 3.5.3.

## Supplementary information

Supplementary information (figures S1-S3; tables S1-S10)

Appendix 1

## Data Availability

The DNA sequence of the novel mtDNA haplotype CM-A76 has been deposited in GenBank (accession number: MN481527).
