# Supplementary material for: Population recovery changes population composition at a major southern Caribbean juvenile developmental habitat for the green turtle, Chelonia mydas
Source: Sci Rep. 2019 Oct 7;9:14392. doi: 10.1038/s41598-019-50753-5 (PMC6779738; doi:10.1038/s41598-019-50753-5)
Supplement: Supplementary file 1 — Supplementary information (figures S1-S3; tables S1-S10) [file 41598_2019_50753_MOESM1_ESM.pdf]

SUPPLEMENTARY INFORMATION for:

Population recovery changes population composition at a major southern Caribbean juvenile developmental habitat for the green turtle, Chelonia mydas

Contents: Figures S1-S3; Tables S1-S10.

Authors and affiliations
Jurjan P. van der Zee[1,2*], Marjolijn J.A. Christianen[1,3], Mabel Nava[4], Ximena Velez-Zuazo[4,5], Wensi Hao[1], Martine Bérubé[1,6], Hanneke van Lavieren[7], Michael Hiwat[7], Rachel Berzins[8], Johan Chevalier[9], Damien Chevallier[10], Marie-Clélia Lankester[9], Karen A. Bjorndal[11], Alan B. Bolten[11], Leontine E. Becking[2,12]† and Per J. Palsbøll[1,6]†

*Author for correspondence (email: j.p.van.der.zee@rug.nl)
†Shared senior authorship

[1]Marine Evolution and Conservation, Groningen Institute for Evolutionary Life Sciences, University of Groningen, Nijenborg 7 9747 AG Groningen, the Netherlands
[2]Wageningen Marine Research, Ankerpark 27 1781 AG Den Helder, the Netherlands
[3]Aquatic Ecology and Water Quality Management Group, Wageningen University & Research, P.O. Box 47, 6700 AA Wageningen, the Netherlands
[4]Sea Turtle Conservation Bonaire, P.O. Box 492, Kaya Korona 53 Kralendijk, Bonaire, the Caribbean Netherlands
[5]Smithsonian Conservation Biology Institute, National Zoological Park, Washington DC, USA
[6]Center for Coastal Studies, 5 Holway Avenue, Provincetown, MA 02657, USA
[7]WWF Guianas, Henck Arronstraat 63, Paramaribo, Suriname
[8]ONCFS Guyane, Campus Agronomique, BP316, 97379 Kourou, French Guiana
[9]RNN Amana, Réserve Naturelle de l'Amana, Maison de la Réserve, 270 Avenue 31 Décembre, 97319 Awala-Yalimapo, French Guiana
[10]Université de Strasbourg, CNRS, IPHC, UMR 7178, Strasbourg, France
[11]Archie Carr Center for Sea Turtle Research and Department of Biology, University of Florida, Gainesville, FL 32611, USA
[12]Marine Animal Ecology Group, Wageningen University & Research, P.O. Box 338, 6700 AH Wageningen, the Netherlands

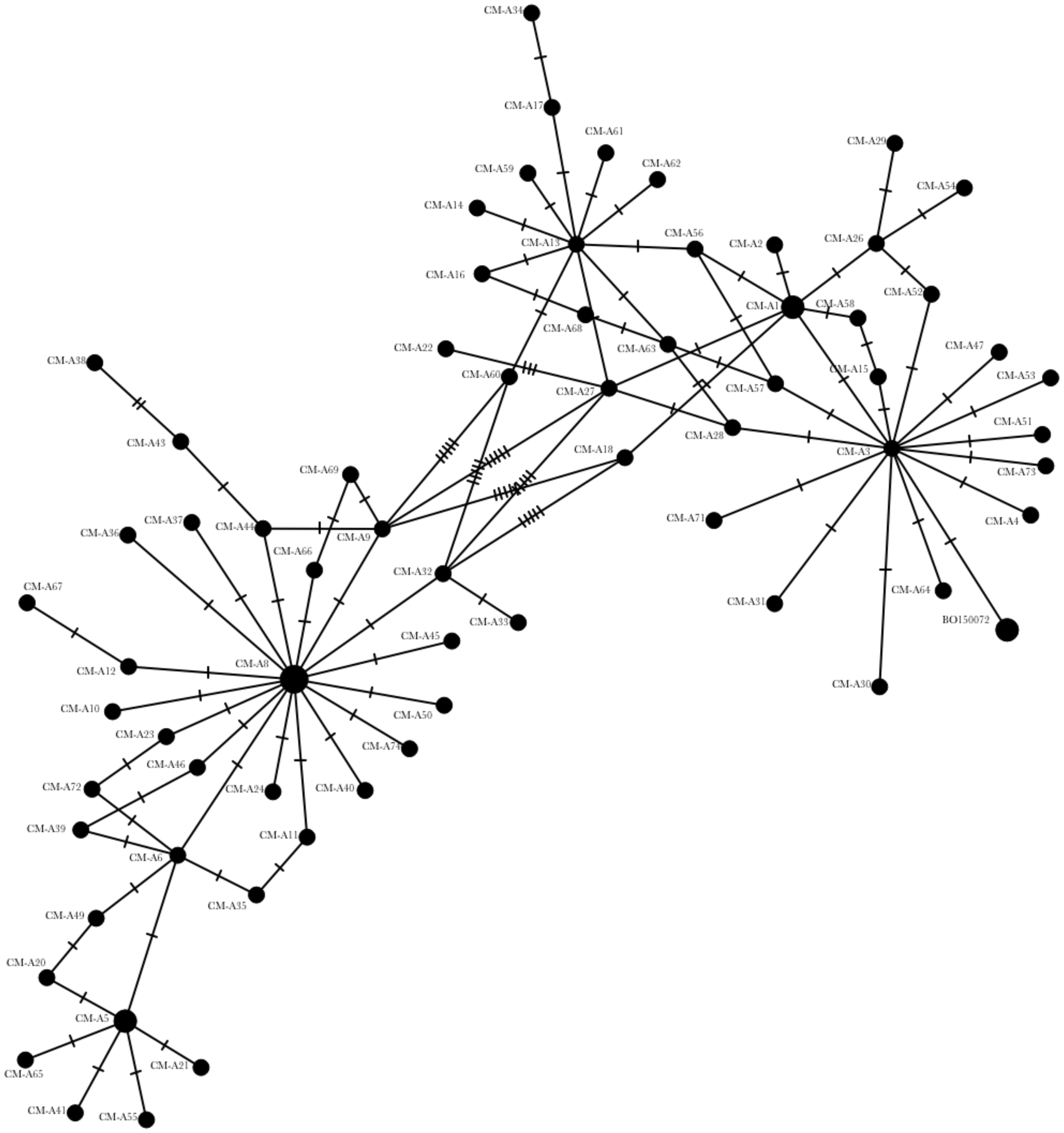

Figure S1. Minimum spanning network showing known Atlantic green turtle mtDNA haplotypes and the novel haplotype CM-A076 (sample 'BO150072') identified in our study. The number of mutations between haplotypes is indicated by the number of perpendicular lines between different nodes.

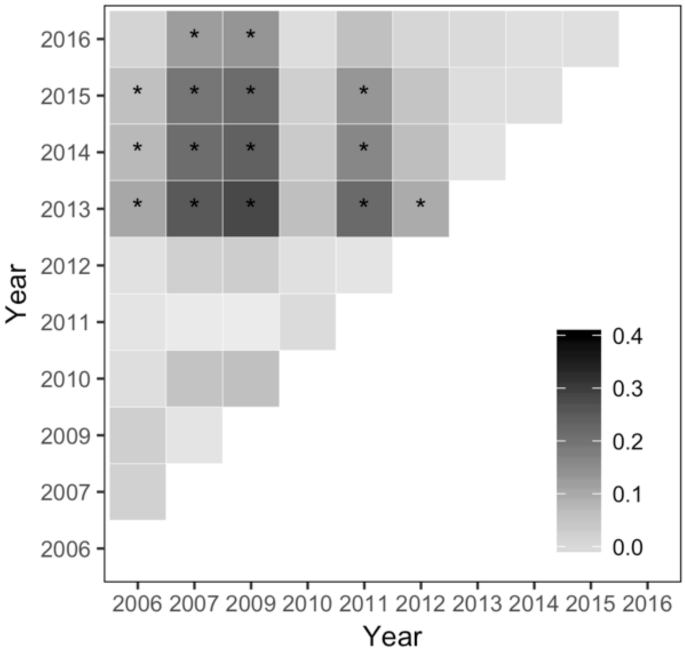

Figure S2. Heat map of pairwise estimates of ϕ_{ST} among years (* denotes P < 0.05) for all juveniles (<75.0cm SCL).

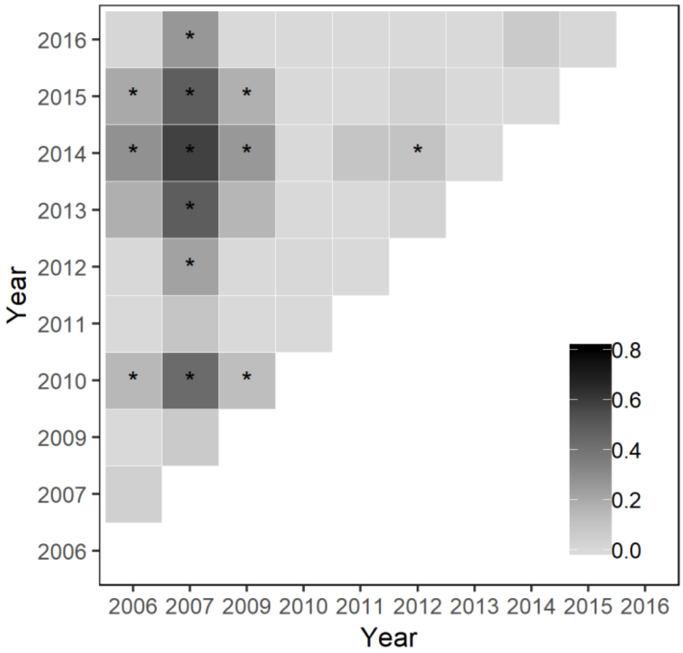

Figure S3. Heat map of pairwise estimates of ϕ_{ST} among years (* denotes P < 0.05) for small juveniles (SCL <50.0cm).

Table S1. Haplotype counts per year, for the temporal samples 2006-2007 and 2015-2016 and for the total data for all juveniles (<75.0cm SCL). Recaptures were excluded in the total data counts (i.e. the number of unique individuals per size category was counted). Haplotype counts for small juveniles (<50.0cm SCL) are shown between parentheses.

Haplotype	2006	2007	2009	2010	2011	2012	2013	2014	2015	2016	2006/2007	2015/2016	Total
CM-A01	0 (0)	1 (0)	1 (1)	5 (3)	1 (1)	1 (1)	4 (2)	6 (3)	11 (6)	4 (2)	1 (0)	14 (8)	30 (18)
CM-A02	1 (0)	0 (0)	0 (0)	0 (0)	0 (0)	0 (0)	0 (0)	1 (1)	0 (0)	0 (0)	1 (0)	0 (0)	2 (1)
CM-A03	18 (7)	15 (3)	9 (7)	25 (19)	5 (1)	17 (12)	16 (6)	24 (15)	51 (23)	13 (4)	33 (10)	61 (27)	173 (89)
CM-A04	0 (0)	0 (0)	0 (0)	0 (0)	0 (0)	1 (0)	0 (0)	0 (0)	1 (0)	0 (0)	0 (0)	1 (0)	1 (0)
CM-A05	10 (6)	19 (8)	14 (8)	15 (5)	4 (1)	9 (5)	3 (1)	6 (1)	15 (4)	6 (3)	29 (14)	21 (7)	90 (41)
CM-A06	1 (1)	0 (0)	0 (0)	0 (0)	1 (0)	1 (0)	0 (0)	0 (0)	1 (0)	0 (0)	1 (1)	1 (0)	3 (1)
CM-A08	3 (1)	1 (1)	0 (0)	1 (0)	0 (0)	2 (1)	0 (0)	1 (1)	2 (2)	0 (0)	4 (2)	2 (2)	10 (6)
CM-A13	0 (0)	0 (0)	0 (0)	0 (0)	0 (0)	0 (0)	0 (0)	0 (0)	1 (1)	0 (0)	0 (0)	1 (1)	1 (1)
CM-A16	0 (0)	0 (0)	0 (0)	1 (0)	0 (0)	0 (0)	0 (0)	0 (0)	1 (0)	1 (1)	0 (0)	2 (1)	3 (1)
CM-A17	2 (1)	0 (0)	0 (0)	0 (0)	0 (0)	0 (0)	0 (0)	0 (0)	0 (0)	0 (0)	2 (1)	0 (0)	2 (1)
CM-A18	0 (0)	0 (0)	2 (2)	0 (0)	0 (0)	0 (0)	1 (1)	1 (1)	2 (0)	1 (1)	0 (0)	3 (1)	5 (5)
CM-A20	1 (0)	0 (0)	0 (0)	0 (0)	0 (0)	1 (1)	0 (0)	0 (0)	0 (0)	0 (0)	1 (0)	0 (0)	2 (1)
CM-A21	0 (0)	0 (0)	0 (0)	0 (0)	1 (0)	0 (0)	0 (0)	0 (0)	0 (0)	0 (0)	0 (0)	0 (0)	1 (0)
CM-A22	1 (0)	0 (0)	0 (0)	0 (0)	0 (0)	0 (0)	0 (0)	0 (0)	0 (0)	0 (0)	1 (0)	0 (0)	1 (0)
CM-A28	0 (0)	0 (0)	0 (0)	0 (0)	0 (0)	0 (0)	0 (0)	0 (0)	1 (0)	0 (0)	0 (0)	1 (0)	1 (0)
CM-A29	0 (0)	1 (0)	0 (0)	1 (0)	0 (0)	0 (0)	0 (0)	0 (0)	0 (0)	0 (0)	1 (0)	0 (0)	2 (0)
CM-A47	1 (0)	0 (0)	0 (0)	0 (0)	0 (0)	0 (0)	0 (0)	0 (0)	0 (0)	1 (1)	1 (0)	1 (1)	2 (1)
CM-A64	0 (0)	0 (0)	0 (0)	0 (0)	0 (0)	1 (1)	0 (0)	0 (0)	1 (0)	0 (0)	0 (0)	1 (0)	1 (1)
CM-A76	0 (0)	0 (0)	0 (0)	0 (0)	0 (0)	0 (0)	0 (0)	1 (1)	2 (2)	0 (0)	0 (0)	2 (2)	2 (2)
Total	38 (16)	37 (12)	26 (18)	48 (27)	12 (3)	33 (21)	24 (10)	40 (23)	89 (38)	26 (12)	75 (28)	111 (50)	332 (169)

Table S2. Sample size (*n*), number of haplotypes (*k*), haplotype diversity (*h*) and nucleotide diversity (*π*) per year, and for 2006-2007 and 2015-2016.

Year	*n*	*k*	*h*	*π*
2006	38	9	0.72 ± 0.06	0.011 ± 0.070
2007	37	5	0.59 ± 0.05	0.011 ± 0.073
2009	26	4	0.61 ± 0.07	0.011 ± 0.074
2010	48	6	0.63 ± 0.05	0.010 ± 0.066
2011	12	5	0.76 ± 0.09	0.011 ± 0.078
2012	33	8	0.67 ± 0.07	0.011 ± 0.070
2013	24	4	0.53 ± 0.10	0.005 ± 0.039
2014	40	7	0.61 ± 0.08	0.007 ± 0.046
2015	89	12	0.63 ± 0.05	0.008 ± 0.050
2016	26	6	0.70 ± 0.07	0.009 ± 0.057
2006-2007	75	11	0.66 ± 0.04	0.011 ± 0.072
2015-2016	111	13	0.65 ± 0.04	0.008 ± 0.052

Table S3. Sample size (*n*), number of haplotypes (*k*), mean haplotype diversity (*h*) and nucleotide diversity (*π*) per year, and for 2006-2007 and 2015-2016 for small juveniles (<50.0cm SCL).

Year	*n*	*k*	*h*	*π*
2006	16	5	0.70 ± 0.08	0.011 ± 0.076
2007	12	3	0.53 ± 0.14	0.008 ± 0.061
2009	18	4	0.67 ± 0.07	0.011 ± 0.075
2010	27	3	0.48 ± 0.10	0.007 ± 0.047
2011	3	3	1.00 ± 0.27	0.014 ± 0.096
2012	21	6	0.64 ± 0.10	0.010 ± 0.067
2013	10	4	0.64 ± 0.15	0.005 ± 0.039
2014	23	7	0.57 ± 0.12	0.004 ± 0.030
2015	38	6	0.61 ± 0.08	0.006 ± 0.042
2016	12	6	0.85 ± 0.07	0.010 ± 0.064
2006-2007	28	5	0.64 ± 0.06	0.011 ± 0.071
2015-2016	50	9	0.67 ± 0.06	0.007 ± 0.046

Table S4. Estimates of pairwise ϕ_{ST} (below diagonal) and P-values (above diagonal) among years. Significant estimates ($P < 0.05$) are highlighted in bold.

	2006	2007	2009	2010	2011	2012	2013	2014	2015	2016
2006		0.174	0.17	0.588	0.622	0.881	**0.027**	**0.03**	**0.025**	0.255
2007	0.019		0.857	0.068	0.841	0.16	**0.002**	**0.001**	**0**	**0.022**
2009	0.025	-0.031		0.063	0.772	0.179	**0.001**	**0.001**	**0.001**	**0.031**
2010	-0.014	0.052	0.061		0.378	0.68	0.079	0.104	0.095	0.472
2011	-0.033	-0.051	-0.053	-0.006		0.536	**0.03**	**0.042**	**0.03**	0.158
2012	-0.024	0.022	0.029	-0.019	-0.031		**0.041**	0.066	0.058	0.271
2013	**0.103**	**0.251**	**0.28**	0.062	**0.217**	**0.097**		0.797	0.531	0.354
2014	**0.073**	**0.213**	**0.236**	0.036	**0.162**	0.064	-0.027		0.855	0.54
2015	**0.058**	**0.194**	**0.214**	0.025	**0.133**	0.049	-0.011	-0.015		0.699
2016	0.011	**0.121**	**0.134**	-0.012	0.058	0.006	-0.002	-0.017	-0.018	

Table S5. Estimates of pairwise ϕ_{ST} (below diagonal) and P-values (above diagonal) among years for small juveniles (<50.0cm SCL). Significant estimates ($P < 0.05$) are highlighted in bold.

	2006	2007	2009	2010	2011	2012	2013	2014	2015	2016
2006		0.174	0.17	0.588	0.622	0.881	**0.027**	**0.03**	**0.025**	0.255
2007	0.019		0.857	0.068	0.841	0.16	**0.002**	**0.001**	**0**	**0.022**
2009	0.025	-0.031		0.063	0.772	0.179	**0.001**	**0.001**	**0.001**	**0.031**
2010	-0.014	0.052	0.061		0.378	0.68	0.079	0.104	0.095	0.472
2011	-0.033	-0.051	-0.053	-0.006		0.536	**0.03**	**0.042**	**0.03**	0.158
2012	-0.024	0.022	0.029	-0.019	-0.031		**0.041**	0.066	0.058	0.271
2013	**0.103**	**0.251**	**0.28**	0.062	**0.217**	**0.097**		0.797	0.531	0.354
2014	**0.073**	**0.213**	**0.236**	0.036	**0.162**	0.064	-0.027		0.855	0.54
2015	**0.058**	**0.194**	**0.214**	0.025	**0.133**	0.049	-0.011	-0.015		0.699
2016	0.011	**0.121**	**0.134**	-0.012	0.058	0.006	-0.002	-0.017	-0.018	

Table S6. Number of counted nests between 2001 and 2017 for French Guiana and Suriname.

Year	French Guiana	Suriname
2001	-	5781
2002	799	10648
2003	774	4905
2004	1566	9240
2005	524	4828
2006	2406	13852
2007	747	9908
2008	3158	18235
2009	1767	10850
2010	4551	30568
2011	2822	6520
2012	2066	8063
2013	4516	42720
2014	1001	6003
2015	2228	18462
2016	770	10096
2017	2318	35343

Table S7. Multiple linear regression and model selection results. Results are shown per step in the stepwise algorithm. Model parameters are shown per year and size class. AIC (Akaike Information Criterion) values are reported for each parameter set, as well as *P*-values for each included parameter. Significant estimates ($P < 0.05$) are highlighted in bold.

Year	Size	Step	Parameters	AIC	$P(\log(N))$	$P(D)$	$P(Nr)$
2006-2007	<75.0cm	1	$\log(N) + D + Nr$	-32.47	0.189	0.119	0.942
2006-2007	<75.0cm	2	$\log(N) + D$	-37.44	**0.047**	0.094	-
2015-2016	<75.0cm	1	$\log(N) + D + Nr$	-32.47	0.464	0.348	0.316
2015-2016	<75.0cm	2	$D + Nr$	-33.75	-	0.428	**0.020**
2015-2016	<75.0cm	3	Nr	-34.97	-	-	0.012
2006-2007	<50.0cm	1	$\log(N) + D + Nr$	-41.81	0.600	0.086	0.860
2006-2007	<50.0cm	2	$\log(N) + D$	-43.77	0.527	0.070	-
2006-2007	<50.0cm	3	D	-45.27	-	0.066	-
2015-2016	<50.0cm	1	$\log(N) + D + Nr$	-17.46	0.478	0.318	0.589
2015-2016	<50.0cm	2	$\log(N) + D$	-19.07	0.633	0.367	-
2015-2016	<50.0cm	3	D	-20.79	-	0.360	-
2015-2016	<50.0cm	4	-	-21.82	-	-	-

Table S8. MCMC parameters for Bayesian mixed stock analysis. Multiple chains were run for each analysis where the starting contribution from one of the putative sources was set to 0.95, while the remaining 0.05 was divided equally among the remaining sources. A sampling interval of 1 meant each iteration (of the 50,000 remaining iterations after burn-in) was sampled.

Parameters	Regions	Rookeries
MCMC chains	4	18
MCMC total chain length	100,000	100,000
Burn-in	50,000	50,000
MCMC chain sampling interval	1	1

Table S9. Annual population growth rates (*r*) at nest sites in the north-western and south-western Caribbean from Mazaris et al. (2017) that were used in the present study. The Palm Beach County growth rate was estimated as the weighted average of growth rates estimated for nest sites within Palm Beach County (see table S5). Estimates of adult female abundance (*N*) were obtained from Seminoff et al. (2015). See the *References* section in the main text for details.

Site	*r*	*N*
Tortuguero, Costa Rica	0.017	131751
Guanal, Cuba	0.080	124
San Felipe, Cuba	0.163	162
Campeche, Mexico	0.171	2207
Quintana Roo, Mexico	0.114	18257
Yucatan, Mexico	0.132	2111
Brevard County (ACNWR), Florida, USA	0.183	3979
Broward County, Florida, USA	0.084	157
Palm Beach County, Florida, USA	0.117	2006
Rocas Atoll, Brazil	0.024	275
Bioko Island, Equatorial Guinea	-0.088	850

Table S10. Annual population growth rates (*r)* and the number of recorded nests (no. nests) at nest sites within Palm Beach County from Mazaris *et al*. (2017). Nest counts were obtained from

http://discover.pbcgov.org/erm/Publications/SeaTurtleNestingDensitiesAreaBeaches.pdf.

Site	*r*	No. nests
Boca Raton beaches	0.102	116
Delray	0.087	18
Gulfstream (includes Gulfstream Park)	0.135	29
Highland	0.051	105
Jupiter Inlet Colony	0.094	81
Jupiter/Carlin	0.111	176
Jupiter/Juno	0.086	667
MacArthur Park	0.074	227
Manalapan (includes Ritz Carlton)	0.247	89
Palm Beach Midtown (includes Breaker's)	0.134	9
Singer Island (includes Ocean Reef Park)	0.103	228
Tequesta/Coral Cove	0.178	437
